# Smoking is significantly associated with increased risk of COVID-19 and other respiratory infections

**DOI:** 10.1038/s42003-021-02685-y

**Published:** 2021-10-28

**Authors:** Daniel B. Rosoff, Joyce Yoo, Falk W. Lohoff

**Affiliations:** 1 Section on Clinical Genomics and Experimental Therapeutics, National Institute on Alcohol Abuse and Alcoholism, National Institutes of Health, Bethesda, MD USA; 2 NIH-Oxford-Cambridge Scholars Program; Nuffield Department of Population Health, University of Oxford, Oxford, UK

**Keywords:** Influenza virus, Risk factors, Addiction

## Abstract

Observational studies suggest smoking, cannabis use, alcohol consumption, and substance use disorders (SUDs) may impact risk for respiratory infections, including coronavirus 2019 (COVID-2019). However, causal inference is challenging due to comorbid substance use. Using summary-level European ancestry data (>1.7 million participants), we performed single-variable and multivariable Mendelian randomization (MR) to evaluate relationships between substance use behaviors, COVID-19 and other respiratory infections. Genetic liability for smoking demonstrated the strongest associations with COVID-19 infection risk, including the risk for very severe respiratory confirmed COVID-19 (odds ratio (OR) = 2.69, 95% CI, 1.42, 5.10, *P*-value = 0.002), and COVID-19 infections requiring hospitalization (OR = 3.49, 95% CI, 2.23, 5.44, *P*-value = 3.74 × 10^−8^); these associations generally remained robust in models accounting for other substance use and cardiometabolic risk factors. Smoking was also strongly associated with increased risk of other respiratory infections, including asthma-related pneumonia/sepsis (OR = 3.64, 95% CI, 2.16, 6.11, *P*-value = 1.07 × 10^−6^), chronic lower respiratory diseases (OR = 2.29, 95% CI, 1.80, 2.91, *P*-value = 1.69 × 10^−11^), and bacterial pneumonia (OR = 2.14, 95% CI, 1.42, 3.24, *P*-value = 2.84 × 10^−4^). We provide strong genetic evidence showing smoking increases the risk for COVID-19 and other respiratory infections even after accounting for other substance use behaviors and cardiometabolic diseases, which suggests that prevention programs aimed at reducing smoking may be important for the COVID-19 pandemic and have substantial public health benefits.

## Abstract (web summary)

Rosoff, Yoo, and Lohoff perform a large-scale genetic epidemiological analysis using Mendelian Randomization to evaluate possible relationships between smoking and substance use behaviours and risk of respiratory infections. They observed a highly significant association between smoking and several respiratory infections, including COVID-19 infection risk.

## Introduction

Since the first reported cases in Wuhan, China in December 2019^1^, coronavirus disease 2019 (COVID-19) has subsequently affected more than 200 countries and continues to be a global pandemic of substantial worldwide morbidity and mortality^2,3^. More broadly, upper and lower respiratory infections (URIs and LRIs, respectively) and other respiratory diseases (i.e., asthma, chronic obstructive pulmonary disease (COPD), etc.) are leading causes of yearly worldwide morbidity and mortality^4,5^. For example, the Global Burden of Disease Study estimated that LRIs caused more than two million deaths globally in 2016^4^, while approximately 2.3 million people died from COPD in 2015^5^. Respiratory infection and diseases are also a large economic burden: URIs result in more than 40 million missed days of school and work per year^6^.

Substance use (tobacco smoking, cannabis use, and alcohol consumption) are risk factors linked with adverse lung and respiratory outcomes^7–9^. For example, observational data has shown chronic heavy alcohol consumption to be associated with increased risk for pneumonia^7^ and acute respiratory distress syndrome^10^, while cannabis smoke has been shown to contain many of the same toxins and irritants as smoke derived from tobacco^11^, but may differ from tobacco in its association with bronchitis and other respiratory infections^12^. In addition, it has been suggested that chronic alcohol abuse may compromise the ability of immune cells to destroy bacteria in the lungs, which may result in an increased vulnerability to respiratory infections like pneumonia and tuberculosis^13^.

Paralleling the COVID-19 pandemic have been increases in substance use^14^, which combined with data showing approximately 10.8% of US adults suffering from a substance use disorder (SUD)^15^ and recent work using electronic health records (EHRs) to show that individuals with a SUD are at increased risk for COVID-19^16^, suggest identifying potential causal relationships between substance use, SUD and respiratory infectious diseases would have substantial public health benefits.

However, observational studies cannot be used to reliably identify causality due to limitations such as residual confounding and reverse causality^17^. For example, outcomes reached from observational studies may be subject to unmeasured confounders like comorbid disorders or underlying genetic differences that may lead to biased estimates, and consequently, may not reflect true causal relationships^18,19^. While randomized controlled trials (RCTs) are considered the “gold standard”, RCTs can be both unethical and impractical^20,21^. Constructing an RCT to examine the effect of substance use on respiratory infection risk may be further complicated by other existing comorbidities.

Mendelian randomization (MR) is a genetic approach that uses genetic variants as instrumental variables to explore causal relations between exposures (e.g., alcohol consumption, tobacco smoking, cannabis use) and health outcomes (e.g., respiratory infections and diseases). This technique takes publicly available genome wide association studies to screen for suitable genetic instrumental variables, which allows researchers to perform MR studies without the need to recruit new patients^22^. Because germline variants are randomly assorted at meiosis, MR may be considered conceptually equivalent to RCTs, though a more naturalized version^19,22^. More specifically, given genetic instruments cannot be influenced by other confounders (i.e., lifestyle, or environmental factors), MR studies, are in theory, less susceptible to confounding or reverse causality than traditional observational studies^23^. Therefore, MR is an important analytical approach to strengthen causal inference when RCTs are challenging due to methodological or ethical constraints^24^. Given the potential for confounding and limited causal inference derived from observational data, we used large, publicly available genome-wide association study (GWAS) data and two-sample MR methods to evaluate the relationships between substance use, substance use disorders (cannabis use disorder (CUD) and alcohol use disorder (AUD)) and respiratory infection and disease outcomes. Finding the genetic liability for smoking increases the risk for COVID-19 and several other respiratory infections, even after accounting for other substance use behaviors builds upon recent literature identifying modifiable risk factors for COVID-19 risk^9,25,26^, and also may inform research and clinical practice given the recent increase in substance use, abuse, and use disorders paralleling the COVID-19 pandemic^14^.

## Results

### Associations of substance use and SUDs with COVID-19 infection risk

COVID-19 results comparing SVMR and MVMR results are presented in Table 1. Supplementary Data 8–12 present the full COVID-19 results. Broadly, among all substance use exposures, the genetic liability for lifetime tobacco smoking consistently demonstrated the strongest associations with COVID-19 infection risk, including the risk for very severe respiratory confirmed COVID-19 (SVMR odds ratio (OR) = 2.69, 95% CI, 1.42, 5.10, *P*-value = 0.002), and also the risk for COVID-19 infection requiring hospitalization (hospitalized COVID-19 vs population: SVMR odds ratio (OR) = 3.49, 95% CI, 2.23, 5.44, *P*-value = 3.74 × 10^−8^; MVMR accounting for substance use disorders OR = 3.61, 95% CI, 2.19, 5.95, *P*-value = 4.92 × 10^−7^; and hospitalized vs not hospitalized COVID-19: SVMR OR = 3.44, 95% CI, 1.72, 6.87, *P*-value = 4.60 × 10^−^^4^; MVMR OR = 3.61, 95% CI, 1.63, 8.01, *P*-value = 0.002) (Table 1; Supplementary Data 8, 10, and 11). This association remained robust in secondary sensitivity analyses excluding UK Biobank participants in the COVID-19 outcome GWAS, but with reduced precision (hospitalized COVID-19 vs population: SVMR OR = 2.42,95% CI, 1.46, 4.01, *P*-value = 6.09 × 10^−4^; MVMR OR = 2.62, 95% CI, 1.46, 4.71, *P*-value = 0.001; and hospitalized vs not hospitalized COVID-19: SVMR OR = 3.27, 95% CI, 1.15, 9.33, *P*-value = 0.03; MVMR OR = 4.84, 95% CI, 1.46, 15.39, *P*-value = 0.008) (Supplementary Data 8, 10, and 11). Importantly, these associations were consistent across complementary SVMR and MVMR methods, including single variable GSMR (Supplementary Data 8, 10, and 11). Leave-one-out analyses highlight variants with heterogeneous causal effects that would be flagged as invalid by MR PRESSO and MV MR Lasso and removed for outlier corrected results (Supplementary Data 9).

**Table 1:** Single variable and multivariable MR results of the genetic liability of alcohol, cannabis and lifetime smoking exposures on COVID-19 outcomes.

	Single-variable MR					Multivariable MR				
	N SNPs	OR	95% CI Lower	95% CI Upper	*P*-value	N SNPs	MV OR	95% CI Lower	95% CI Upper	*P*-value
*Very severe respiratory confirmed COVID-19 vs. population*										
Tobacco smoking	91	2.69	1.42	5.10	**0.002**	111	2.72	1.27	5.82	0.010
Cannabis use	28	1.17	0.92	1.50	0.207	114	1.03	0.76	2.80	0.856
CUD	22	0.97	0.82	1.15	0.748	111	1.02	0.86	1.22	0.805
Drinks per week	22	1.39	0.52	3.74	0.511	114	0.83	0.33	2.30	0.698
AUD	9	0.91	0.75	1.11	0.344	111	0.95	0.82	1.09	0.442
*Hospitalized COVID-19 vs. not hospitalized COVID-19*										
Tobacco smoking	91	3.44	1.72	6.87	**4.60E**−**04**	111	3.61	1.63	8.01	**0.002**
Cannabis use	28	0.87	0.68	1.11	0.270	114	1.02	0.77	2.78	0.883
CUD	21	1.07	0.91	1.25	0.404	111	1.04	0.87	1.25	0.627
Drinks per week	21	0.69	0.27	1.76	0.432	114	0.37	0.15	1.45	0.034
AUD	9	0.83	0.69	0.99	0.035	111	0.94	0.81	1.10	0.451
*Hospitalized COVID-19 vs. population*										
Tobacco smoking	91	3.49	2.23	5.44	**3.74E**−**08**	111	3.61	2.19	5.95	**4.92E**−**07**
Cannabis use	28	0.99	0.85	1.16	0.887	114	1.00	0.83	2.73	0.964
CUD	21	0.99	0.90	1.10	0.871	111	1.01	0.90	1.13	0.915
Drinks per week	21	1.01	0.57	1.81	0.964	114	0.59	0.32	1.80	0.079
AUD	8	0.94	0.82	1.08	0.375	111	0.98	0.89	1.07	0.633
*COVID-19 vs. population*										
Tobacco smoking	91	1.21	0.97	1.52	0.095	111	1.25	0.95	1.64	0.104
Cannabis use	28	1.11	1.02	1.21	0.022	114	1.04	0.95	2.83	0.404
CUD	21	0.98	0.93	1.03	0.436	111	0.98	0.92	1.04	0.411
Drinks per week	21	1.09	0.81	1.47	0.565	114	0.93	0.68	2.52	0.622
AUD	8	1.08	1.00	1.16	0.039	111	1.03	0.98	1.08	0.310

Notes: Results from two sample SVMR inverse-variance weighted MR analysis; outliers identified by MR PRESSO global test and, for MVMR, MV MR Lasso penalization were removed; estimated associations reported as odds ratios with 95% confidence intervals. Boldface indicates statistical significance after correction for multiple comparisons (*P* < 0.0025). Genetic instruments selected from five GWASs, selection threshold *P* < 5 × 10^−8^ or *P* < 5 × 10^−6^ (CUD and AUD), clumped at linkage disequilibrium (LD) *r*^*2*^ = 0.001 (10 000 kilobase pair window); N SNPs differs across outcomes depending on number of genetic instruments found in outcome GWASs. *CUD* cannabis use disorder, *AUD* alcohol use disorder, *COVID-19* coronavirus 2019, *MR* Mendelian randomization, *GWAS* genome wide association study, *N SNPs* number of single-nucleotide polymorphism (genetic instruments), *OR* odds ratio, *CI* confidence interval.

Given the strong associations of lifetime tobacco smoking and COVID-19 risk, we further evaluated robustness by performing MVMR analyses accounting for cardiometabolic disorders (CAD, T2D, and obesity) previously reported as risk factors for COVID-19 risk^27–29^. Genetic liability for lifetime tobacco smoking generally remained associated with increased risk for COVID-19 hospitalization (e.g., accounting for CAD, hospitalized COVID-19 vs. population: MVMR OR = 3.18, 95% CI, 2.06, 4.92, *P*-value = 1.80 × 10^−7^; accounting for Type 2 diabetes, MVMR OR = 4.16, 95% CI, 2.51, 6.92, *P*-value = 3.76 × 10^−8^; accounting for obesity, MVMR OR = 3.75, 95% CI, 2.25, 6.25, *P*-value = 4.01 × 10^−7^) (Supplementary Data 12).

### Associations of substance use and SUDs with other respiratory infectious disease risk

We further assessed the genetic relationships between substance use and respiratory infections. Tables 2 and 3 compares SVMR and MVMR results for asthma-related respiratory infections, bronchitis, and the common cold; Tables 4 and 5 compares SVMR and MVMR results for influenza and pneumonias. Supplementary Data 13–17 contain the full FinnGen results.

**Table 2:** Single variable and multivariable MR results of the genetic liability of alcohol, cannabis and lifetime smoking exposures on asthma-related respiratory infections.

Outcome	Exposure	Single-variable MR					Multivariable MR				
		N SNPs	OR	95% CI Lower	95% CI Upper	*P*-value	N SNPs	OR	95% CI Lower	95% CI Upper	*P*-value
*Asthma related acute respiratory infections*											
	Tobacco smoking	116	2.06	1.39	3.05	3.15E−04	137	2.07	1.31	3.26	0.002
	Cannabis Use	35	1.07	0.98	1.16	0.128	142	1.03	0.88	1.22	0.704
	CUD	27	1.10	1.03	1.19	0.007	137	1.01	0.92	1.11	0.835
	Drinks Per Week	32	0.92	0.56	1.53	0.757	142	1.06	0.65	1.72	0.816
	AUD	11	1.04	0.93	1.17	0.456	137	1.01	0.93	1.10	0.826
*Asthma related infections*											
	Tobacco smoking	115	2.31	1.61	3.31	5.69E−06	127	2.15	1.45	3.17	1.21E−04
	Cannabis Use	35	1.02	0.94	1.09	0.672	142	1.06	0.91	1.23	0.484
	CUD	27	1.08	1.00	1.16	0.040	127	0.98	0.91	1.07	0.662
	Drinks Per Week	32	0.94	0.61	1.44	0.773	142	0.96	0.61	1.49	0.845
	AUD	11	1.04	0.95	1.13	0.385	127	1.03	0.96	1.10	0.358
*Asthma-related pneumonia*											
	Tobacco smoking	116	2.52	1.59	3.97	7.29E−05	138	3.64	2.16	6.11	1.07E−06
	Cannabis Use	36	0.95	0.85	1.06	0.382	136	1.07	0.90	1.27	0.500
	CUD	27	1.07	0.98	1.18	0.120	138	0.95	0.85	1.06	0.380
	Drinks Per Week	31	1.47	0.83	2.59	0.187	136	1.09	0.61	1.93	0.272
	AUD	11	1.09	0.97	1.21	0.132	138	0.99	0.90	1.08	0.755
*Asthma-related pneumonia or sepsis*											
	Tobacco smoking	116	2.54	1.61	4.02	6.54E−05	138	3.66	2.17	6.16	1.04E−06
	Cannabis Use	35	0.96	0.87	1.07	0.472	141	1.08	0.90	1.31	0.401
	CUD	27	1.07	0.98	1.18	0.122	138	0.95	0.85	1.07	0.399
	Drinks Per Week	32	1.85	1.04	3.26	0.035	141	1.15	0.66	2.00	0.616
	AUD	11	1.09	0.97	1.21	0.138	138	0.98	0.89	1.08	0.734

Notes: Results from two sample SVMR inverse-variance weighted MR analysis; outliers identified by MR PRESSO global test and, for MVMR, MV MR Lasso penalization were removed; estimated associations reported as odds ratios with 95% confidence intervals. Boldface indicates statistical significance after correction for multiple comparisons (*P* < 0.000714). Genetic instruments selected from 5 GWASs, selection threshold *P* < 5 × 10^−8^ or *P* < 5 × 10^−6^ (CUD and AUD), clumped at linkage disequilibrium (LD) *r*^*2*^ = 0.001 (10 000 kilobase pair window); N SNPs differs across outcomes depending on number of genetic instruments found in outcome GWASs. *CUD* cannabis use disorder, *AUD* alcohol use disorder, *MR* Mendelian randomization, *GWAS* genome wide association study, *N SNPs* number of single-nucleotide polymorphism (genetic instruments), *OR* odds ratio, *CI* confidence interval.

**Table 3:** Single variable and multivariable MR results of the genetic liability of alcohol, cannabis and lifetime smoking exposures on chronic obstructive pulmonary disorder, bronchitis, and the common cold.

Outcome	Exposure	Single-variable MR					Multivariable MR				
		N SNPs	OR	95% CI Lower	95% CI Upper	*P*-value	N SNPs	OR	95% CI Lower	95% CI Upper	*P*-value
*COPD (Kela code 203)*											
	Tobacco smoking	117	2.08	1.49	2.88	1.35E−05	121	2.07	1.55	2.77	7.34E−07
	Cannabis Use	35	1.02	0.96	1.08	0.531	126	1.00	0.91	1.10	0.998
	CUD	27	1.03	0.99	1.08	0.178	121	0.98	0.92	1.04	0.440
	Drinks Per Week	31	0.76	0.53	1.09	0.130	126	0.84	0.62	1.15	0.099
	AUD	11	1.00	0.94	1.06	0.959	121	1.00	0.95	1.05	0.948
*Bronchitis*											
	Tobacco smoking	117	1.66	0.75	3.66	0.210	138	1.43	0.58	3.55	0.436
	Cannabis Use	36	0.93	0.79	1.10	0.405	142	1.04	0.76	1.40	0.820
	CUD	27	1.07	0.90	1.27	0.453	138	1.04	0.85	1.26	0.713
	Drinks Per Week	33	1.12	0.44	2.86	0.806	142	1.63	0.66	3.98	0.286
	AUD	11	1.03	0.84	1.25	0.783	138	1.09	0.92	1.28	0.325
*Acute nasopharyngitis (common cold)*											
	Tobacco smoking	117	1.80	0.98	3.32	0.058	138	1.76	0.86	3.59	0.119
	Cannabis Use	36	1.01	0.87	1.16	0.941	142	1.15	0.89	1.48	0.273
	CUD	27	1.03	0.91	1.17	0.640	138	1.09	0.93	1.27	0.276
	Drinks Per Week	33	1.04	0.49	2.21	0.916	142	0.58	0.28	1.24	0.160
	AUD	11	0.97	0.82	1.14	0.684	138	0.96	0.85	1.10	0.571

Notes: Results from two sample SVMR inverse-variance weighted MR analysis; outliers identified by MR PRESSO global test and, for MVMR, MV MR Lasso penalization were removed; estimated associations reported as odds ratios with 95% confidence intervals. Boldface indicates statistical significance after correction for multiple comparisons (*P* < 0.000714). Genetic instruments selected from 5 GWASs, selection threshold *P* < 5 × 10^−8^ or *P* < 5 × 10^−6^ (CUD and AUD), clumped at linkage disequilibrium (LD) *r*^*2*^ = .001 (10 000 kilobase pair window); N SNPs differs across outcomes depending on number of genetic instruments found in outcome GWASs. *CUD* cannabis use disorder, *AUD* alcohol use disorder, *COPD* chronic obstructive pulmonary disorder, *MR* Mendelian randomization, *GWAS* genome wide association study, *N SNPs* number of single-nucleotide polymorphism (genetic instruments), *OR* odds ratio, *CI* confidence interval.

**Table 4:** Single variable and multivariable MR results of the genetic liability of alcohol, cannabis and lifetime smoking exposures on influenza, chronic lower respiratory diseases, and acute upper respiratory infections.

Outcome	Exposure	Single-variable MR					Multivariable MR				
		N SNPs	OR	95% CI Lower	95% CI Upper	*P*-value	N SNPs	OR	95% CI Lower	95% CI Upper	*P*-value
*Influenza*											
	Tobacco smoking	117	1.70	1.09	2.65	0.019	138	1.71	1.01	2.91	0.048
	Cannabis Use	36	1.01	0.91	1.13	0.836	142	1.17	0.97	1.41	0.093
	CUD	27	1.03	0.95	1.13	0.455	138	1.01	0.90	1.13	0.891
	Drinks Per Week	33	1.16	0.67	1.99	0.599	142	0.74	0.43	1.27	0.276
	AUD	11	1.08	0.95	1.24	0.240	138	1.00	0.91	1.10	0.955
*Influenza and Pneumonia*											
	Tobacco smoking	116	1.53	1.25	1.87	**4.13E**−**05**	138	1.62	1.27	2.05	**8.50E**−**05**
	Cannabis Use	35	1.01	0.97	1.06	0.587	137	1.05	0.97	1.13	0.261
	CUD	27	1.05	1.01	1.09	0.013	138	0.99	0.94	1.04	0.662
	Drinks Per Week	33	0.97	0.75	1.25	0.812	137	0.97	0.77	1.22	0.780
	AUD	11	1.06	1.01	1.11	0.021	138	1.00	0.96	1.05	0.955
*Chronic Lower Respiratory Diseases*											
	Tobacco smoking	113	2.23	1.73	2.87	**5.69E**−**10**	122	2.29	1.80	2.91	**1.69E**−**11**
	Cannabis Use	35	1.04	0.99	1.09	0.172	121	1.02	0.94	1.10	0.986
	CUD	27	1.05	1.01	1.10	0.015	122	1.02	0.97	1.07	0.533
	Drinks Per Week	31	0.91	0.69	1.20	0.492	121	0.89	0.68	1.15	0.020
	AUD	11	1.00	0.95	1.06	0.906	122	1.01	0.97	1.05	0.678
*Acute Upper Respiratory Infections*											
	Tobacco smoking	116	1.32	1.09	1.61	0.004	130	1.47	1.19	1.82	**3.52E**−**04**
	Cannabis Use	34	1.02	0.97	1.06	0.484	135	1.06	0.99	1.14	0.115
	CUD	27	1.04	1.00	1.09	0.035	130	1.02	0.98	1.07	0.346
	Drinks Per Week	33	0.93	0.74	1.18	0.568	135	0.95	0.77	1.18	0.636
	AUD	11	0.99	0.95	1.04	0.699	130	0.98	0.95	1.02	0.355

Notes: Results from two sample SVMR inverse-variance weighted MR analysis; outliers identified by MR PRESSO global test and, for MVMR, MV MR Lasso penalization were removed; estimated associations reported as odds ratios with 95% confidence intervals. Boldface indicates statistical significance after correction for multiple comparisons (*P* < 0.000714). Genetic instruments selected from 5 GWASs, selection threshold *P* < 5 × 10^−8^ or *P* < 5 × 10^−6^ (CUD and AUD), clumped at linkage disequilibrium (LD) *r*^*2*^ = .001 (10 000 kilobase pair window); N SNPs differs across outcomes depending on number of genetic instruments found in outcome GWASs. *CUD* cannabis use disorder, *AUD* alcohol use disorder, *MR* Mendelian randomization, *GWAS* genome wide association study, *N SNPs* number of single-nucleotide polymorphism (genetic instruments), *OR* odds ratio, *CI* confidence interval.

As with COVID-19 infection risk results, we found that the genetic liability of lifetime tobacco smoking was the substance use risk factor with the strongest associations, including results that were robust in MVMR models. Tobacco smoking, for example, was associated with increased risk of asthma-related infections and asthma-related pneumonia/sepsis (SVMR OR = 2.52, 95% CI, 1.59, 3.97, *P*-value = 7.29 × 10^−7^; accounting for substance use disorders, MVMR OR = 3.64, 95% CI, 2.16, 6.11, *P*-value = 1.07 × 10^−^^6^), but for neither bronchitis nor the common cold (Table 3; Supplementary Data 13–15). Tobacco smoking was also associated with chronic lower respiratory diseases (SVMR OR = 2.23, 95% CI, 1.73, 2.87, *P*-value = 5.69 × 10^−^^10^; MVMR OR = 2.29, 95% CI, 1.80, 2.91, *P*-value = 1.69 × 10^−^^11^) and several pneumonia-related outcomes, including bacterial pneumonia (SVMR OR = 2.22, 95% CI, 1.57, 3.15, *P*-value = 7.32 × 10^−^^6^; MVMR OR = 2.14, 95% CI, 1.42, 3.24, *P*-value = 2.84 × 10^−^^4^) (Table 5, Supplementary Data 13–15).

**Table 5:** Single variable and multivariable MR results of the genetic liability of alcohol, cannabis and lifetime smoking exposures on pneumonia risk.

Outcome	Exposure	Single-variable MR					Multivariable MR				
		N SNPs	OR	95% CI Lower	95% CI Upper	*P*-value	N SNPs	OR	95% CI Lower	95% CI Upper	*P*-value
*Bacterial pneumonia*											
	Tobacco smoking	117	2.22	1.57	3.15	**7.32E**−**06**	138	2.14	1.42	3.24	**2.84E**−**04**
	Cannabis Use	36	1.05	0.96	1.15	0.268	142	0.97	0.84	1.12	0.685
	CUD	27	1.05	0.98	1.13	0.145	138	1.02	0.93	1.12	0.648
	Drinks Per Week	33	1.17	0.72	1.89	0.530	142	1.19	0.78	1.83	0.416
	AUD	11	1.04	0.95	1.14	0.426	138	1.01	0.93	1.08	0.892
*All Pneumoniae*											
	Tobacco smoking	115	1.52	1.22	1.88	**1.34E**−**04**	134	1.46	1.15	1.85	0.002
	Cannabis Use	34	1.01	0.96	1.06	0.833	136	1.03	0.95	1.12	0.439
	CUD	27	1.05	1.01	1.09	0.014	134	0.98	0.94	1.04	0.544
	Drinks Per Week	31	0.97	0.76	1.24	0.814	136	0.96	0.75	1.23	0.750
	AUD	11	1.05	1.00	1.11	0.056	134	1.01	0.97	1.05	0.625
*Viral Pneumonia*											
	Tobacco smoking	117	1.66	0.53	5.17	0.386	138	1.70	0.44	6.52	0.443
	Cannabis Use	36	1.07	0.81	1.41	0.638	142	1.34	0.84	2.15	0.221
	CUD	27	1.00	0.78	1.28	0.993	138	0.90	0.67	1.20	0.479
	Drinks Per Week	33	2.24	0.60	8.43	0.232	142	1.27	0.32	5.04	0.736
	AUD	11	1.13	0.85	1.51	0.409	138	1.09	0.85	1.39	0.501

Notes: Results from two sample SVMR inverse-variance weighted MR analysis; outliers identified by MR PRESSO global test and, for MVMR, MV MR Lasso penalization were removed; estimated associations reported as odds ratios with 95% confidence intervals. Boldface indicates statistical significance after correction for multiple comparisons (*P* < 0.000714). Genetic instruments selected from 5 GWASs, selection threshold *P* < 5 × 10^−8^ or *P* < 5 × 10^−6^ (CUD and AUD), clumped at linkage disequilibrium (LD) *r*^*2*^ = .001 (10 000 kilobase pair window); N SNPs differs across outcomes depending on number of genetic instruments found in outcome GWASs. *CUD* cannabis use disorder, *AUD* alcohol use disorder, *MR* Mendelian randomization, *GWAS* genome wide association study, *N SNPs* number of single-nucleotide polymorphism (genetic instruments), *OR* odds ratio, *CI* confidence interval.

As with the smoking-COVID-19 findings, we tested robustness of the smoking-respiratory infection risk results using additional MVMR models that accounted for cardiometabolic disorders (CAD, T2D, and obesity) with evidence for an impact on respiratory infection risk^30–32^. Our smoking-related results were broadly robust to inclusion of cardiometabolic confounders (Supplementary Data 16). These associations were generally consistent across complementary SVMR and MVMR methods, including single variable GSMR (Supplementary Data 13–16). Leave-one-out analyses again highlight variants with heterogeneous causal effects that would be flagged as invalid by MR PRESSO and MV MR Lasso and removed for outlier corrected results (Supplementary Data 17).

## Discussion

Using large summary-level GWAS data and complementary two-sample MR methods, we show that the genetic liability for tobacco smoking has potential causal relationships with several respiratory infection and disease outcomes, including COVID-19. These tobacco smoking-respiratory findings were supported by multivariable MR analyses accounting for alcohol and cannabis use and abuse, which in addition to the broadly consistent IVW results (within the IVW MR 95% confidence interval but typically less precise) with estimates from the weighted median, weighted mode, and MR Egger sensitivity analyses strengthen causal inference. Further, in single variable MR, we identify potential adverse impacts of CUD on lower respiratory infections, the common cold, and several asthma-related infections, suggesting evidence for a dose-dependent impact of cannabis use where heavy cannabis use may be harmful to the respiratory system. In parallel, we find little evidence for an alcohol-respiratory infection relationship suggesting that previous observational data may be due to confounding.

Our COVID-19 results extend recent MR studies showing adverse effects of smoking on COVID-19 risk by accounting for highly comorbid alcohol consumption, cannabis use, and SUDs, which when combined with reports suggesting smoking intensifies the severity of COVID-19 symptoms^33,34^, the risk for being admitted to an intensive care unit or requiring ventilation^34^, and recent transcriptomics-based work showing that smoking may increase the expression of angiotensin converting enzyme 2 (*ACE2*), the putative receptor for severe acute respiratory syndrome coronavirus 2 (SARS-CoV-2) (the virus that causes COVID-19)^35^, suggests smoking may be an important modifiable risk factor for COVID-19 risk.

Our genetics-based findings support and extend the observational literature identifying tobacco smoking as a risk factor for respiratory infection and diseases^9,25,26^, and add to the recent MR literature identifying potential causal links of smoking with reduced lung function^36^, lung cancer^37^, and increased mortality due to respiratory disease^38^. Potential mechanisms by which smoking increases respiratory infection risk include structural changes to the respiratory tract and a dysregulated cellular and humoral immune response, including peribronchiolar inflammation, decreased levels of circulating immunoglobulins, and changes to pathogen adherence. For example, smoking has been shown to stimulate the release of catecholamine and corticosteroids, which may, in turn, increase circulating CD8^+^ lymphocytes and suppress the host defense against infections. Notably, many immunological effects related to smoking may resolve within six weeks of smoking cessation, which suggests that smoking cessation programs may have an important impact on reducing respiratory infections.

Regarding cannabis use, while we failed to find evidence of any relationships, smoking cannabis, like tobacco smoking, may prompt the onset of coughing, which could consequently increase viral transmission, or may possibly exacerbate respiratory symptoms.

As cannabis is the most used drug worldwide—an estimated 188 million recreational users worldwide—this aspect of cannabis use may have important implications for the spread of COVID-19. In contrast, the single-variable MR CUD results demonstrated adverse effects on several respiratory outcomes, but not COPD, which supports the existing literature^39–41^; however, accounting for lifetime tobacco smoking attenuated the CUD results, thus highlighting the complex nature of these relationships. Further, habitual cannabis smoking may have several effects on respiratory and immune systems that may impact respiratory infection susceptibility. For example, structural abnormalities in alveolar macrophages and coincident dysregulated cytokine production and antimicrobial activity have been reported. While our study provides preliminary genetic evidence suggesting potential causal relationships between heavy cannabis use and respiratory infection, additional triangulating lines of evidence (i.e., immune monitoring studies) are required to further elucidate the CUD-respiratory infection relationship. However, given that the toxin and irritant profiles of cannabis and tobacco smoke are similar^11^, the direct route of administration via inhalation for these substances could result in dysregulated pulmonary physiology which may, in turn, increase infection risk.

In contrast to our tobacco smoking findings, we failed to find genetic evidence of respiratory implications due to alcohol consumption not meeting the threshold of AUD, or binge drinking, suggesting that previous observational literature may be due to confounding from other comorbid behaviors—such as smoking—that may be the true causal risk factors for respiratory infections. For example, observational and genetic evidence have shown a strong association between alcohol consumption and smoking. It has been estimated that 85% of smokers consume alcohol^42–44^ and alcohol drinkers are 75% more likely than abstainers to smoke^45^. Therefore, it is possible that the observational study-based alcohol-respiratory infection links may be due, instead, to tobacco smoking; however, future work will be needed to confirm this hypothesis. In addition, it is important to note that our results should not be interpreted as suggesting that alcohol does not impact overall lung health and structure, which has been previously reported^7^. Further, while we failed to find evidence that weekly alcohol consumption impacted COVID-19 risk, the Centers for Disease Control recently showed that dining at on-site locations, such as restaurants and bars, is associated with increased COVID-19 risk; since alcohol consumption may lower inhibition and increase impulsivity, individuals consuming alcohol may take social distancing less seriously, and thereby unintentionally spread the SARS-CoV-2 virus.

This study has several strengths including the use of multiple alcohol consumption and cannabis use variables, which enabled us to evaluate various dimensions of substance use and abuse and identify possible causal relationships of substance use disorders and respiratory outcomes. In addition, our main single variable analyses included multiple MR methods, each relying on orthogonal assumptions, which provide confidence in robustness of the results and strengthen causal inference^46^. Our multivariable two-sample MR design, the most appropriate design given the strong correlation between tobacco smoking, alcohol consumption and cannabis use, yielded estimates that account for these correlated behaviors for each exposure on COVID-19 risk and other respiratory outcomes. Another strength is our extension of MVMR to test the robustness of the main tobacco smoking findings by incorporating other potential confounders that may impact infectious disease risk (obesity, cardiovascular disease, and T2D).

This study also has limitations. A main limitation is the possibility of collider bias—especially with regards to the COVID-19 datasets^47^. Collider bias may occur when analyses are controls or selects the sample based upon a collider variable that is caused by both the exposure and outcome variables and distorts the true underlying association^48,49^. The recent commentaries by Griffith et al. (2020) and Tattan-Birch et al. (2020) discuss in detail the potential for collider bias in COVID-19 datasets^47,49^, and are important for context when interpreting COVID-19 findings based upon observational data. For example, an observational study from early in the COVID-19 pandemic reported an apparent protective effect of tobacco smoking on COVID-19 risk^50^; however, as Tattan-Birch et al. discuss, both smoking and COVID-19 may cause coughing, which, during the COVID-19 pandemic, may increase the likelihood for smokers to be tested and their subsequent overrepresentation among clinical study participants testing negative for COVID-19^49^. As a result, among samples with COVID-19 tests, smoking may appear to have a protective effect^49^. While it is often not possible to ensure the absence of collider bias^47^, we aimed to design our study incorporating measures that may mitigate its impact. For example, we used the most recently released version of publicly available COVID-19 data (from January 18, 2021)^51^ that may include participants more representative of the general population compared to samples collected earlier in the COVID-19 pandemic. Reassuringly, we also found similar smoking effect estimates in several respiratory-related infection outcomes, which suggests a broader impact of smoking on the respiratory system that extends to COVID-19.

In addition, as with all self-reported substance use literature, these exposures may be either under- or over-reported^52^. Because many of the datasets included UK Biobank participants, who are more educated, lead healthier lifestyles, and have fewer health problems than the UK population^53^, this discrepancy may limit the applicability of our findings to other populations. Regarding our mainly null alcohol-respiratory infection results, it is possible that alcohol may have indirect impacts on infection risk through a modified immune response^54^, or other system dysregulations that may modulate infection risk that we were not able to directly assess using MR. However, like other recent psychiatric MR studies where the exposure instruments included a relaxed statistical threshold, our binge drinking and AUD instruments were comprised of independent SNPs associated with the respective drinking behavior (i.e., *P*-value < 5 × 10^−6^) for SNP inclusion due to the lack of conventionally GWS SNPs (*P*-value < 5 × 10^−8^)^55,56^, which may impact the results. Because heavy alcohol consumption and AUD have been previously linked with acute respiratory distress syndrome^10^—one of the most severe complications of COVID-19—future studies should re-evaluate the links between heavy alcohol consumption and AUD when better powered GWAS data become available.

Further, the included samples were comprised of primarily white individuals of European ancestry, and research has shown strong racial, ethnic, and socioeconomic disparities in COVID-19 risk and severity^57–59^. Therefore, we caution the generalization of these findings and urge future work to investigate these relationships using a genetics-based approach in other populations when the data become available. Another limitation is the overlap of the UKB participants between the alcohol consumption, lifetime smoking, and COVID-19 outcomes, which may bias resulting estimates^60^. However, potential bias would likely be minimal^60^, and it has also been shown that two-sample MR may be used in single samples provided the data is derived from large biobanks, i.e., the UKB, FinnGen, etc^61^. Also, results were largely unchanged when we performed analyses using the COVID-19 endpoints excluding UKB participants suggesting minimal bias.

In conclusion, our data provide genetic evidence of adverse relationships between smoking and many respiratory-related disease outcomes ranging from the common cold to severe COVID-19, which suggests prevention programs aimed at smoking cessation and prevention may have public health and clinical benefits.

## Methods

### Data sources and genetic instruments

**Fig. 1:**
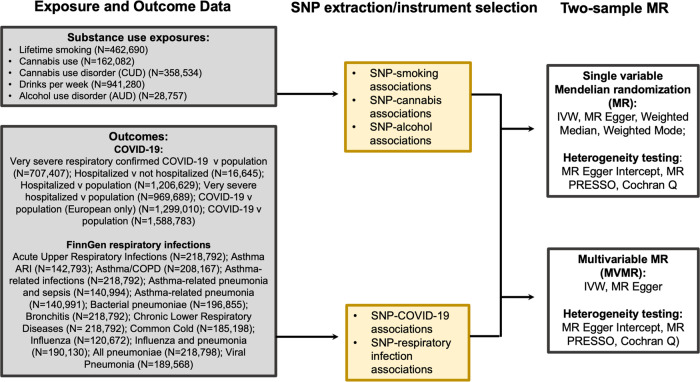
Study overview. Abbreviations: SNP: single nucleotide polymorphism; COVID-19: coronavirus disease 2019; COPD: Chronic obstructive pulmonary disease; IVW, Inverse Variance Weighted MR; SVMR; single variable Mendelian randomization; GSMR: generalized summary Mendelian randomization; MVMR: multivariable Mendelian randomization; MR PRESSO: MR pleiotropy residual sum and outlier; HEIDI: heterogeneity in dependent instruments.

Summary-level data for both modifiable risk factor instrument and infectious disease outcome data were derived from publicly available GWASs in populations of predominantly European ancestry (Fig. 1; Table 6; Supplementary Data 1). All GWASs have existing ethical permissions from their respective institutional review boards and include participant informed consent with rigorous quality control. For this study, we included all exposure SNPs associated at conventional genome-wide significance (GWAS) *P* < 5 × 10^−8^ for smoking, alcohol and cannabis use, and 5 × 10^−6^ for AUD and CUD due to the relatively low number of SNPs at GWS, clumped at linkage disequilibrium (LD) *r*^*2*^ = 0.001 and a distance of 10,000 kb, using reference samples comprised of participants of European ancestry ^62^.

**Table 6:** Study data sources.

Phenotypes:	Consortium	First author (Year)	Sample size	*N* cases	*N* controls	Population
***Exposures:***						
Drinks per week	GSCAN	Liu (2019)	941,280	NA	NA	European
Alcohol use disorder	PGC	Walters (2019)	28,757	8485	20,272	European
Lifetime smoking	MRC-IEU	Wootton (2019)	462,690	NA	NA	European
Cannabis Use	PGC	Pasman (2018)	162,082	43,380	118,702	European
Cannabis Use Disorder	PGC	Johnson (2020)	358,534	14,808	343,726	European
Coronary Artery Disease	CARDIoGRAMplusC4D	van der Harst (2017)	547,261	122,733	424,528	European
Type 2 Diabetes	DIAGRAM	Xue (2018)	590,283	50,721	539,562	European
Obesity Class I	GIANT	Berndt (2013)	98,697	32,858	65,839	European
Obesity Class II	GIANT	Berndt (2013)	725,46	9889	62,657	European
Obesity Class III	GIANT	Berndt (2013)	50,364	2896	47,468	European
***COVID-19 outcomes (Round 5):***						
Very severe respiratory confirmed COVID-19 vs. population	COVID 19-hg	—	707,407	4606	702,801	European
Very severe respiratory confirmed COVID-19 vs. population (excluding UKB)	COVID 19-hg	—	378,521	4297	374,224	European
Hospitalized vs. not hospitalized COVID-19	COVID 19-hg	—	16,645	4829	11,816	European
Hospitalized vs. not hospitalized COVID-19 (excluding UKB)	COVID 19-hg	—	10,365	3159	7,206	European
Hospitalized COVID-19 vs. population	COVID 19-hg	—	1,206,629	9373	1,197,256	European
Hospitalized COVID-19 vs. population (excluding UKB)	COVID 19-hg	—	876,382	7703	868,679	European
COVID-19 vs. population	COVID 19-hg	—	1,588,783	29,071	1,559,712	European
COVID-19 vs. population (excluding UKB)	COVID 19-hg	—	1,25,3716	22,581	1,231,135	European
***FinnGen outcomes (Release 5):***						
Acute Upper Respiratory Infections	FinnGen	—	218,792	35,847	182,945	European
Asthma related acute respiratory infections	FinnGen	—	142,793	7348	135,445	European
Asthma/COPD (Kela code 203)	FinnGen	—	208,167	21,444	186,723	European
Asthma related infections	FinnGen	—	218,792	58,925	159,867	European
Asthma-related pneumonia or sepsis	FinnGen	—	140,994	5545	135,449	European
Asthma-related pneumonia	FinnGen	—	140,981	5532	135,449	European
Bacterial pneumonia	FinnGen	—	196,855	7987	188,868	European
Bronchitis	FinnGen	—	218,792	27,361	191,431	European
Chronic Lower Respiratory Diseases	FinnGen	—	218,792	32,069	186,723	European
Acute nasopharyngitis (common cold)	FinnGen	—	185,198	2253	182,945	European
Influenza	FinnGen	—	193,130	4262	188,868	European
Influenza and Pneumonia	FinnGen	—	218,792	29,924	188,868	European
All Pneumoniae	FinnGen	—	218,798	27,376	191,422	European
Viral Pneumonia	FinnGen	—	189,568	700	188,868	European

*GSCAN* GWAS & Sequencing Consortium of Alcohol and Nicotine, *MRC-IEU* Medical Research Council Integrative Epidemiology Unit, *PGC* Psychiatric Genomics Consortium, *GIANT* Genetic Investigation of ANthropometric Traits, *CARDIoGRAMplusC4D* Coronary ARtery DIsease Genome-wide Replication and Meta-analysis (CARDIoGRAM) plus The Coronary Artery Disease (C4D) Genetics), *DIAGRAM* DIAbetes Genetics Replication and Meta-analysis, *COVID-19* coronavirus disease 2019.

### Tobacco smoking

We included lifetime smoking instruments from the recent GWAS of a lifetime smoking index/score (which combined smoking initiation, duration, heaviness and cessation), conducted in a sample of 462 690 current, former and never smokers in the UKB (mean score value 0.359 (standard deviation (SD) = 0.694); sample: 54% female, mean age 56.7 years, 54% never smokers, 36% former smokers, and 11% current smokers^63,64^. (An SD increase in lifetime smoking index score would be equivalent to smoking 20 cigarettes per day for 15 years and stopping 17 years previously or 60 cigarettes per day for 13 years and stopping 22 years previously)^63^ (Supplementary Data 2).

### Cannabis use

We included two cannabis-related instrument sets: cannabis use and CUD. Summary statistics for lifetime cannabis use (a yes/no variable of whether participants reported using cannabis during their lifetime) were obtained from the PGC meta-analysis GWAS of 3 cohorts (International Cannabis Consortium (35,297 respondents, 55.5 percent female, ages 16–87, mean 35.7 years; 42.8 percent had used cannabis); UKB (126 785 respondents, 56.3 percent female, ages 39–72, mean age 55.0 years, 22.3 percent had used cannabis); and 23andMe (22,683 respondents, 55.3 percent female, ages 18–94, mean age 54.0 years, 43.2% had used cannabis))^65,66^. CUD instruments were obtained from a recent PGC meta-analysis of three cohorts of predominantly European ancestry (PGC, Lundbeck Foundation Initiative for Integrative Psychiatric Research (iPSYCH), and deCODE cohorts, excluding related individuals from PGC family-based cohorts; demographics not available), including 14,808 cases of cannabis abuse or dependence defined as meeting DSM-IIIR, DSM-IV, DSM-5, or ICD10 codes (depending on study cohort) criteria; the 358 534 controls were defined as anyone not meeting the criteria^67,68^ (Supplementary Data 3).

### Alcohol consumption

We included two instrument sets related to alcohol use: drinks per week^69^, and AUD. Drinks per week instruments were obtained from the GSCAN GWAS meta-analysis of 29 cohorts (941 280 individuals; demographics not available) of predominantly white European ancestry^69,70^. Given the varied cohort methods used to measure alcohol consumption (binned, normalized, etc.), the data was log transformed: thus, the effect estimate is measured in log transformed drinks per week^69^ (Supplementary Data 4). For the AUD instrument set, we used the Psychiatric Genomics Consortium (PGC) GWAS meta-analysis of 28 cohorts (51.6% female, 8485 cases, 20,657 controls) of predominantly European ancestry^71,72^. AUD was diagnosed by either clinician rating or semi-structured interview using DSM-IV criteria including the presence of at least three of seven alcohol-related symptoms (withdrawal, drinking larger amounts/drinking for longer time, tolerance, desire or attempts to cut down drinking, giving up important activities to drink, time related to drinking, or continued alcohol consumption despite psychological and/or physical problems)^73^ (Supplementary Data 4).

For the multivariable MR (MVMR) analyses, we concatenated independent instrument sets for alcohol use, cannabis use and lifetime smoking, and also AUD, CUD, and lifetime smoking, clumping the resulting two multivariable (MV) instrument sets to exclude intercorrelated SNPs with pairwise LD *r*^*2*^ > 0.001, leaving 141 and 126 MV instruments, respectively (Supplementary Data 5 and 6).

Obesity, coronary heart disease (CAD), and Type 2 Diabetes (T2D) have been identified as risk factors for COVID-19^27–29^, and other respiratory infections^30–32^. Therefore, in supplementary sensitivity analyses to further test robustness of the lifetime smoking results, we concatenated independent instrument sets for lifetime smoking and, alternatively, CAD using the CARDIoGRAMplusC4D-UK Biobank CAD (Coronary ARtery DIsease Genome wide Replication and Meta-analysis (CARDIoGRAM) plus The Coronary Artery Disease (C4D) Genetics) GWAS meta-analysis^74,75^; T2D, using a recent meta-analysis of three T2D studies, i.e. DIAbetes Genetics Replication and Meta-analysis (DIAGRAM), Genetic Epidemiology Research on Aging (GERA) and the full cohort release of UKB^76,77^; and obesity, using GWASs from GIANT (Genetic Investigation of ANthropometric Traits)^78,79^ (see Supplementary Data 1 for more information; Supplementary Data 7).

*F* statistics for the unconditional instruments were strong (>10, Supplementary Data 2–4). We were unable to calculate conditional *F* statistics to assess the strength of the multivariable instrument sets: SVMR statistical methods recently extended to two sample MVMR are appropriate only for non-overlapping exposure summary level data sources. When overlapping, the requisite pairwise covariances between SNP associations are only determinable by using individual level data ^80^.

### COVID-19 outcomes

We used summary GWAS statistics from the COVID-19 Host Genetics Initiative (COVID-19 hg) meta-analysis round 5a (18 January 2021 release date) (https://www.covid19hg.org/results)^81^ for four COVID-19 phenotypes in cohorts of European ancestry, both including and excluding the UKB cohorts for sensitivity analyses (N cases; N controls): very severe respiratory confirmed COVID-19 versus population (4606; 702,801); very severe respiratory confirmed COVID-19 versus population excluding UKB cohorts (4297; 374,224); hospitalized versus not hospitalized COVID-19 (4829; 11,816); hospitalized versus not hospitalized COVID-19 excluding UKB cohorts (3159; 7206); hospitalized COVID-19 versus population (9373; 1,197,256); hospitalized COVID-19 versus population excluding UKB cohorts (7703; 868,679); COVID-19 versus population (29,071; 1,559,712); COVID-19 versus population excluding UKB cohorts (22,581; 1,231,135) (demographics not available) (Fig. 1; Table 6; Supplementary Data 1).

### Other respiratory infection and disease outcomes

We used data from FinnGen Release 5 (released to public, 11 May 2021) for additional respiratory-related outcomes^82^, including acute upper respiratory infections, asthma related acute respiratory infections, pneumonia, influenza, bronchitis, chronic lower respiratory diseases, and acute nasopharyngitis (common cold) (*N* ≤ 218,792) (Fig. 1; Table 6; Supplementary Data 1). FinnGen is a public-private partnership incorporating genetic data for disease endpoints from Finnish biobanks and Finnish health registry EHRs^82^. Detailed documentation is provided on the FinnGen website (https://finngen.gitbook.io/documentation/).

### Sample independence

Participant overlap in samples used to estimate genetic associations between exposures and outcomes can increase weak instrument bias (WIB) in MR analyses^60,83^, but to a lesser extent with large biobank samples (including UKB and deCODE). Given the large size of the overlapping cohorts (e.g., UKB, deCode) (Supplementary Data 1) and the strength of the instruments in both directions (*F* statistics > 10; Supplementary Data 2–4), considerable WIB would not be expected^60,84^. We have conducted analyses for COVID-19 outcomes using COVID-19 GWAS performed both including and excluding UKB cohorts.

### Statistics and reproducibility

For SVMR analyses, we used inverse-variance weighted MR (MR IVW) as the main analyses, supplemented by MR-Egger, weighted median, and weighted mode methods. These are complementary robust methods developed to estimate consistent causal effects under weaker assumptions than MR IVW to assess evidence of causal effects for each of alcohol, cannabis and tobacco use, and use disorders on infectious disease outcomes, and evaluate the sensitivity of the analyses to different patterns of violations of IV assumptions^85^. Consistency of results across methods strengthens an inference of causality^85^. For MVMR analyses, we used the multivariable extensions of MR IVW, MR Egger, and MR median ^83,86^.

We used the MR Egger intercept test^87^, Cochran Q heterogeneity test^88^, and multivariable extensions thereof, to evaluate heterogeneity in instrument effects, as heterogeneity may indicate violations of IV assumptions^86,87,89^. The MR pleiotropy residual sum and outlier (MR PRESSO) global test, and multivariable extensions thereof^90^, were used to facilitate identification and removal of outlier instruments to correct potential directional horizontal pleiotropy and resolve detected heterogeneity. For SVMR, we also used, alternatively, Generalized single variable Summary-data based MR (GSMR) to identify and remove instruments with heterogeneous causal estimates suspected to be invalid instruments with apparent pleiotropic effects on both exposure and outcome disease (using the recommended default HEIDI (heterogeneity in dependent instruments) -outlier threshold (0.01) to retain sufficient power to detect heterogeneity)^91^. We used the SVMR Steiger directionality test to test the causal direction between the hypothesized exposure and outcomes^62^. We also performed a leave-one-out analysis to evaluate the potential SNPs within each instrument that may be high influence points^85^.

For MVMR, in addition to the multivariable extension of the MR PRESSO global test, we used the multivariable extension of the MR Lasso method, which applies lasso-type penalization to the direct effects of the instruments on the outcome disease: the so-called post-lasso estimate is obtained by performing MR IVW using only those instruments identified as valid instruments (tuning parameter specified at default heterogeneity stopping rule)^89^. Analyses were carried out using TwoSampleMR, version 0.5.5^85^, MendelianRandomization, version 0.5.0, in the R environment, version 4.0.2; the GSMR method was implemented in the GCTA (Genome-wide Complex Trait Analysis) software (https://cnsgenomics.com/software/gcta/#GSMR).

### Reported results and interpretation of findings

MR IVW odds ratios (OR) with 95% CI, per unit increase in the exposures (e.g., per unit increase of log-transformed alcoholic drinks per week or lifetime smoking index), with *P*-values derived from two-sided tests, corrected for outlier or invalid variants, are presented in Tables 1–5. For our COVID-19 analyses, we used a two-sided α of 0.0025 (based on comparing four COVID-19 outcomes and five substance use exposures) and for the other infectious disease outcomes, a threshold of 0.00071 (based on comparing 14 FinnGen infectious respiratory diseases and five substance use exposures) as a heuristic that allows for follow-up analyses for a plausible number of findings. In assessing consistency and robustness, we looked for estimates substantially agreeing in direction and magnitude (overlapping confidence intervals) across then four complementary MR methods used. We evaluate evidence strength based upon the effect magnitude and direction, the 95% confidence interval of that effect, and the *P-*value.

### Reporting summary

Further information on research design is available in the Nature Research Reporting Summary linked to this article.

## Supplementary information

Description of Additional Supplementary Files

Supplementary Data

Reporting Summary

## Data Availability

All analyses were based upon publicly available data. Single-variable MR and multivariable MR instrument datasets for each substance use behavior required to replicate the findings of this study are available in the Supplemental Data files. Full COVID-19 GWAS summary-level data is available at https://www.covid19hg.org/results/. FinnGen data are available at https://www.finngen.fi/en; lifetime smoking at https://data.bris.ac.uk/data/dataset/10i96zb8gm0j81yz0q6ztei23d; alcohol drinks per week data at: https://genome.psych.umn.edu/index.php/GSCAN; cannabis use disorder and alcohol use disorder data are available through the Psychiatric Genomics Consortium data portal: https://www.med.unc.edu/pgc/download-results/; and the cannabis use data are available through the International Cannabis Consortium at: https://www.ru.nl/bsi/research/group-pages/substance-use-addiction-food-saf/vm-saf/genetics/international-cannabis-consortium-icc/. Coronary artery disease and obesity summary statistics are available through the Cardiovascular Disease Knowledge Portal: https://cvd.hugeamp.org/. Type 2 Diabetes summary-level data is available Type 2 Diabetes Knowledge Portal: https://t2d.hugeamp.org/.

## Code availability

We used TwoSampleMR and MendelianRandomization R packages to perform Mendelian Randomization analysis. These packages are publicly available at https://github.com/MRCIEU/TwoSampleMR, and https://github.com/cran/MendelianRandomization, respectively.
