# Supplementary material for: Smoking is significantly associated with increased risk of COVID-19 and other respiratory infections
Source: Commun Biol. 2021 Oct 28;4:1230. doi: 10.1038/s42003-021-02685-y (PMC8553923; doi:10.1038/s42003-021-02685-y)
Supplement: Supplementary file 1 — Description of Additional Supplementary Files [file 42003_2021_2685_MOESM1_ESM.pdf]

Description of Additional Supplementary Files

File name: Supplementary Data

Description:

Supp Data 1. Phenotype descriptions and sources.

Supp Data 2. Genetic instruments for alcohol use and alcohol use disorder.

Supp Data 3. Genetic instruments for lifetime tobacco smoking.

Supp Data 4. Genetic instruments for cannabis use and cannabis use disorder.

Supp Data 5. Multivariable genetic instruments for alcohol use, cannabis use and lifetime tobacco smoking.

Supp Data 6. Multivariable genetic instruments for alcohol use disorder, cannabis use disorder and lifetime tobacco smoking.

Supp Data 7. Multivariable genetic instruments for lifetime tobacco smoking and coronary artery disease, Type 2 diabetes, and obesity*.

Supp Data 8. Single variable Mendelian randomization analyses of substance use and use disorder on COVID-19.

Supp Data 9. Leave-one-out analyses for substance use and use disorder on COVID-19.

Supp Data 10. Multivariable Mendelian randomization analysis results for alcohol use, cannabis use and lifetime tobacco smoking on COVID-19.

Supp Data 11. Multivariable Mendelian randomization analysis results for alcohol use disorder, cannabis use disorder and lifetime tobacco smoking on COVID-19.

Supp Data 12. Multivariable Mendelian randomization analysis results for lifetime tobacco smoking and, alternatively, coronary artery disease, type 2 diabetes, and obesity on COVID 19.

Supp Data 13. Single variable Mendelian randomization analyses of substance use and use disorder on FinnGen R5 endpoints.

Supp Data 14. Multivariable Mendelian randomization analysis results for substance use on FinnGen R5 endpoints.

Supp Data 15. Multivariable Mendelian randomization analysis results for substance use disorders on FinnGen R5 endpoints.

Supp Data 16. Multivariable Mendelian randomization analysis results for lifetime tobacco smoking and, alternatively, coronary artery disease, type 2 diabetes, and obesity, on FinnGen R5 endpoints.

Supp Data 17. Leave-one-out analyes for substance use and use disorder on FinnGen R5 endpoints.
