# Supplementary material for: Smoking is significantly associated with increased risk of COVID-19 and other respiratory infections
Source: Commun Biol. 2021 Oct 28;4:1230. doi: 10.1038/s42003-021-02685-y (PMC8553923; doi:10.1038/s42003-021-02685-y)
Supplement: Supplementary file 3 — Reporting Summary [file 42003_2021_2685_MOESM3_ESM.pdf]

nature portfolio

Corresponding author(s): Falk W. Lohoff

Last updated by author(s): Aug 10, 2021

Reporting Summary

Nature Portfolio wishes to improve the reproducibility of the work that we publish. This form provides structure for consistency and transparency in reporting. For further information on Nature Portfolio policies, see our Editorial Policies and the Editorial Policy Checklist.

Statistics

For all statistical analyses, confirm that the following items are present in the figure legend, table legend, main text, or Methods section.

n/a	Confirmed	
☐	☒	The exact sample size (n) for each experimental group/condition, given as a discrete number and unit of measurement
☐	☒	A statement on whether measurements were taken from distinct samples or whether the same sample was measured repeatedly
☐	☒	The statistical test(s) used AND whether they are one- or two-sided<br>*Only common tests should be described solely by name; describe more complex techniques in the Methods section.*
☐	☒	A description of all covariates tested
☐	☒	A description of any assumptions or corrections, such as tests of normality and adjustment for multiple comparisons
☐	☒	A full description of the statistical parameters including central tendency (e.g. means) or other basic estimates (e.g. regression coefficient) AND variation (e.g. standard deviation) or associated estimates of uncertainty (e.g. confidence intervals)
☒	☐	For null hypothesis testing, the test statistic (e.g. F, t, r) with confidence intervals, effect sizes, degrees of freedom and P value noted<br>*Give P values as exact values whenever suitable.*
☒	☐	For Bayesian analysis, information on the choice of priors and Markov chain Monte Carlo settings
☒	☐	For hierarchical and complex designs, identification of the appropriate level for tests and full reporting of outcomes
☐	☒	Estimates of effect sizes (e.g. Cohen's d, Pearson's r), indicating how they were calculated

Our web collection on statistics for biologists contains articles on many of the points above.

Software and code

Policy information about availability of computer code

Data collection	This study used publicly available summary-level GWAS data from several sources listed below:<br><br>Full COVID-19 GWAS summary-level data is available at https://www.covid19hg.org/results/. FinnGen data are available at https://www.finngen.fi/en; lifetime smoking at https://data.bris.ac.uk/data/dataset/10i96zb8gm0j81yz0q6ztei23d; alcohol drinks per week data at: https://genome.psych.umn.edu/index.php/GSCAN; frequency of consuming >6 drinks per occasion data are available at: http://www.nealelab.is/uk-biobank; cannabis use disorder and alcohol dependence data are available through the Psychiatric Genomics Consortium data portal: https://www.med.unc.edu/pgc/download-results/; and the cannabis use data are available through the International Cannabis Consortium at: https://www.ru.nl/bsi/research/group-pages/substance-use-addiction-food-saf/vm-saf/genetics/international-cannabis-consortium-icc/
Data analysis	We used TwoSampleMR and MendelianRandomization R packages to perform Mendelian Randomization analysis. These packages are publicly available at https://github.com/MRCIEU/TwoSampleMR, and https://github.com/cran/MendelianRandomization, respectively.

For manuscripts utilizing custom algorithms or software that are central to the research but not yet described in published literature, software must be made available to editors and reviewers. We strongly encourage code deposition in a community repository (e.g. GitHub). See the Nature Portfolio guidelines for submitting code & software for further information.

Data

Policy information about availability of data

All manuscripts must include a data availability statement. This statement should provide the following information, where applicable:

- Accession codes, unique identifiers, or web links for publicly available datasets
- A description of any restrictions on data availability
- For clinical datasets or third party data, please ensure that the statement adheres to our policy

All analyses were based upon publicly available data. Harmonized datasets required to replicate the findings of this study are available in the Supplemental Data files. Full COVID-19 GWAS summary-level data is available at https://www.covid19hg.org/results/. FinnGen data are available at https://www.finngen.fi/en; lifetime smoking at https://data.bris.ac.uk/data/dataset/10i96zb8gm0j81yz0q6ztei23d; alcohol drinks per week data at: https://genome.psych.umn.edu/index.php/GSCAN; frequency of consuming >6 drinks per occasion data are available at: http://www.nealelab.is/uk-biobank; cannabis use disorder and alcohol dependence data are available through the Psychiatric Genomics Consortium data portal: https://www.med.unc.edu/pgc/download-results/; and the cannabis use data are available through the International Cannabis Consortium at: https://www.ru.nl/bsi/research/group-pages/substance-use-addiction-food-saf/vm-saf/genetics/international-cannabis-consortium-icc/

Field-specific reporting

Please select the one below that is the best fit for your research. If you are not sure, read the appropriate sections before making your selection.

- [x] Life sciences
- [ ] Behavioural & social sciences
- [ ] Ecological, evolutionary & environmental sciences

For a reference copy of the document with all sections, see nature.com/documents/nr-reporting-summary-flat.pdf

Life sciences study design

All studies must disclose on these points even when the disclosure is negative.

Sample size	For valid genetic estimates, Mendelian randomization (MR) requires large sample sizes from participants of similar genetic ancestry (Hemani, G, eLife, 2018). Therefore, for our two-sample MR study design, we used the largest available summary-level data from genome-wide associations studies (GWASs) on substance use behaviors and respiratory infections in participants of similar ancestry.
Data exclusions	To improve interpretation and not violate assumptions underlying MR about population stratification, we limited our analyses to data derived from participants of European ancestry. We caution in our limitations section that, as a result of this data exclusion, our findings are limited to this populations and should not be extrapolated to others.
Replication	For all analyses we used several complementary sensitivity analyses to improve causal inference. We also replicated our main COVID-19 findings in a subsetted dataset not including UK Biobank participants
Randomization	Mendelian randomization leverages genetic variation randomly allocated at conception to infer unconfounded genetic estimates
Blinding	The study investigators were blinded to genotyping during data collection.

Reporting for specific materials, systems and methods

We require information from authors about some types of materials, experimental systems and methods used in many studies. Here, indicate whether each material, system or method listed is relevant to your study. If you are not sure if a list item applies to your research, read the appropriate section before selecting a response.

Materials & experimental systems

n/a	Involved in the study	
x		Antibodies
x		Eukaryotic cell lines
x		Palaeontology and archaeology
x		Animals and other organisms
	x	Human research participants
x		Clinical data
x		Dual use research of concern

Methods

n/a	Involved in the study	
x		ChIP-seq
x		Flow cytometry
x		MRI-based neuroimaging

Human research participants

Policy information about studies involving human research participants

Population characteristics	Genetic associations with COVID-19 were taken from eight publicly available meta-analyses published by COVID-19 Host Genetics Initiative (HGI), Round 5, including both males and females, European population, as follows (23&Me cohorts excluded): Very severe respiratory confirmed COVID-19 vs. population (4606 cases, 702801 controls); very severe respiratory confirmed COVID-19 vs. population, excluding UK Biobank (UKB) cohorts (4297 cases, 374224 controls); hospitalized vs. not hospitalized COVID-19 (4829 cases, 11816 controls); hospitalized vs. not hospitalized COVID-19, excluding UKB cohorts (3159 cases, 7206 controls); hospitalized COVID-19 vs. population (9373 cases, 1197256 controls); hospitalized COVID-19 vs. population, excluding UKB cohorts (7703 cases, 868679 controls); COVID-19 vs. population (29071 cases, 1559712 controls); and COVID-19 vs. population, excluding UKB cohorts (22581 cases, 1231135 controls). Genetic associations with other primary respiratory diseases were taken from publicly available GWASs published 11 May 2021 by FinnGen, Release 5, including both males and females, as follows: Acute upper respiratory infections (35847 cases, 182945 controls); asthma related acute respiratory infections (7348 cases, 135445 controls); asthma/COPD (21444 cases, 186723 controls); asthma-related infections (58925 cases, 159867 controls); asthma-related pneumonia or sepsis (5545 cases, 135449 controls); asthma-related pneumonia (5532 cases, 135449 controls); bacterial pneumonia (27361 cases, 191431 controls); bronchitis (27361 cases, 191431 controls); chronic lower respiratory diseases (32069 cases, 186723 controls); acute nasopharyngitis (common cold) (2253 cases, 182945 controls); influenza (4262 cases, 188868 controls); influenza and pneumonia (299924 cases, 188868 controls); all pneumoniae (27376 cases, 181422 controls); and viral pneumoniae (700 cases, 188868 controls). Genetic associations with alcoholic drinks per week were taken from the publicly available meta-analysis published by GWAS & Sequencing Consortium of Alcohol and Nicotine Use (GSCAN) (up to 941280 males and females, combined). Genetic associations with alcohol dependence were taken from the publicly available meta-analysis published by Substance Use Disorder (SUD) group of the Psychiatrics Genomics Consortium (PGC), males and females, combined, European population, only including unrelated individuals from PGC, (8415 cases, 20272 controls). Genetic associations with lifetime smoking were taken from the publicly available GWAS of up to 462690 European population males and females participating in the UKB. Genetic associations with cannabis use were taken from the publicly available meta-analysis published by International Cannabis Consortium (ICC), males and females, combined (43480 cases, 118702 controls). Genetic associations with cannabis use disorder were taken from the publicly available meta-analysis published by PGC, males and females combined, European population, only including unrelated individuals from PGC, Lundbeck Foundation Initiative for Integrative Psychiatric Research (iPSYCH) and deCODE cohorts (14080 cases, 343726 controls). Genetic associations with coronary artery disease were taken from the publicly available meta-analysis of CARDIoGRAMplusC4D and UKB GWASs, males and females, European population, combined (122733 cases, 424528 controls). Genetic associations with Type 2 Diabetes were taken from the publicly available meta-analysis of three studies: DIAbetes Genetics Replication and Meta-analysis (DIAGRAM), Genetic Epidemiology Research on Aging (GERA), and the UKB, males and females, combined, predominantly European population (99.4%), (50721cases, 539562 controls). Genetic associations with obesity were taken from publicly available GWAS published by the Genetic Investigation of ANthropometric Traits (GIANT) consortium, males and females, combined, European population, as follows: obesity class I (32858 cases, 65839 controls); obesity class II (9889 cases, 62657 controls); obesity class III (2896 cases, 47468 controls). Further demographic information may be available in the respective publications of the GWASs
Recruitment	Publicly available GWAS summary statistics were used in this study. For details regarding recruitment for the studies contributing to these GWASs, please see the GWASs publications references, Supp Data 1.
Ethics oversight	All GWASs providing summary statistics used in this study affirmed that ethical approval from the relevant institutional review boards was received; that the GWASs were done in accordance with relevant ethical regulations; and, where applicable, that investigators from each study that may have contributed to the GWAS obtained informed consent from their participants and received ethics approvals from their respective review boards in accordance with applicable regulations. Analysis of publicly available GWAS data or summary statistics used in this study does not require additional ethical approval.

Note that full information on the approval of the study protocol must also be provided in the manuscript.
